# Transcriptome analysis reveals the high ribosomal inhibitory action of 1,4-naphthoquinone on *Meloidogyne luci* infective second-stage juveniles

**DOI:** 10.3389/fpls.2023.1191923

**Published:** 2023-06-05

**Authors:** Joana M. S. Cardoso, Ivânia Esteves, Conceição Egas, Mara E. M. Braga, Hermínio C. de Sousa, Isabel Abrantes, Carla Maleita

**Affiliations:** ^1^ Centre for Functional Ecology - Science for People and The Planet, Department of Life Sciences, University of Coimbra, Coimbra, Portugal; ^2^ Center for Neuroscience and Cell Biology, Faculdade de Medicina, University of Coimbra, Coimbra, Portugal; ^3^ Biocant-Transfer Technology Center, BiocantPark, Cantanhede, Portugal; ^4^ Chemical Process Engineering and Forest Products Research Centre, Department of Chemical Engineering, University of Coimbra, Coimbra, Portugal

**Keywords:** bionematicide, *Meloidogyne*, naphthoquinone, plant-parasitic nematodes, transcriptome

## Abstract

The root-knot nematode (RKN) *Meloidogyne luci* presents a threat to the production of several important crops. This nematode species was added to the European Plant Protection Organization Alert list in 2017. The scarce availability of efficient nematicides to control RKN and the phasing out of nematicides from the market have intensified the search for alternatives, such as phytochemicals with bionematicidal properties. The nematicidal activity of 1,4-naphthoquinone (1,4-NTQ) against *M. luci* has been demonstrated; however, knowledge of the potential mode(s) of action of this compound is still scarce. In this study, the transcriptome profile of *M. luci* second-stage juveniles (J2), the infective stage, in response to 1,4-NTQ exposure was determined by RNA-seq to identify genes and pathways that might be involved in 1,4-NTQ’s mode(s) of action. Control treatments, consisting of nematodes exposed to Tween^®^ 80 (1,4-NTQ solvent) and to water, were included in the analysis. A large set of differentially expressed genes (DEGs) was found among the three tested conditions, and a high number of downregulated genes were found between 1,4-NTQ treatment and water control, reflecting the inhibitory effect of this compound on *M. luci*, with a great impact on processes related to translation (ribosome pathway). Several other nematode gene networks and metabolic pathways affected by 1,4-NTQ were also identified, clarifying the possible mode of action of this promising bionematicide.

## 1 Introduction

Plant-parasitic nematodes (PPNs) are among the most devasting agricultural pests, and from these, root-knot nematodes (RKNs) belonging to the genus *Meloidogyne* are in the top 10 of those with the most ecological and economic impact worldwide. *Meloidogyne incognita*, *M. arenaria*, *M. hapla*, and *M. javanica* are common RKN species, parasitizing almost any species of vascular plants and being widely distributed in agricultural areas around the world (Jones et al., 2013). However, other species, such as *M. luci*, are regarded as of emerging importance. This RKN species was first described in 2014 from isolates from lavender (*Lavandula spica*) collected in Brazil (Carneiro et al., 2014), but reports of *Meloidogyne* sp. females with a similar esterase phenotype have been recorded since 1985 in South America (Argentina, Bolivia, Brazil, Chile, Ecuador, and Guatemala), Iran, and Europe (Turkey and Slovenia) and have been associated with several important plant species (Carneiro et al., 2014; Bellé et al., 2016; Janssen et al., 2016). In 2017, several populations previously identified as *M. ethiopica* in Europe were reclassified, using biochemical and molecular analyses, and identified as *M. luci* (Gerič Stare et al., 2017). *Meloidogyne luci* is recognized as a threat to European countries and has been included in the Alert List of Pest of European and Mediterranean Plant Protection Organization since 2017 (EPPO, 2017). Its presence in Portugal was first reported in 2018 to be associated with potato (*Solanum tuberosum*) (Maleita et al., 2018) and later was found to parasitize tomato (*S. lycopersicum*), the ornamental plant *Cordyline australis*, and the weed *Oxalis corniculata* (Santos et al., 2019). More recently, *M. luci* was also reported in Serbia to be associated with tomato (Bačić et al., 2023).

Over the last 30 years, management of PPN has relied on chemical control with the use of large spectra fumigant and non-fumigant nematicides that have now been largely withdrawn due to their harsh impact on non-target organisms and the environment. To date, only few synthetic target-specific non-fumigant nematicides are available, such as fluensulfone, fluopyram, and fluazaindolizine, but their use on all types of crops is not yet recommended (Oka, 2020). The demand for eco-friendly bionematicides became mandatory for a sustainable agricultural production. During the last few years, new compounds like several plant secondary metabolites have been identified as nematicidal compounds with effects on *Meloidogyne* sp. second-stage juvenile (J2) hatching, paralysis and nematode root attraction, penetration, and reproduction (Aoudia et al., 2012; Ntalli and Caboni, 2012; Aissani et al., 2015; Lu et al., 2017; Sikder and Vestergård, 2020). Moreover, the nematicidal effects of the naturally occurring naphthoquinone (NTQ) compounds, namely, juglone (5-hydroxynaphthalene-1,4-dione; JUG) and 1,4-naphthoquinone (naphthalene-1,4-dione; 1,4-NTQ), have been demonstrated against PPN (Mahajan et al., 1992; Dama, 2002; Esteves et al., 2017; Maleita et al., 2017; Cha et al., 2019; Dama, 2019). In a previous study, 1,4-NTQ induced *M. luci* J2 mortality and inhibited hatching, penetration, and reproduction in tomato (Maleita et al., 2022). Nevertheless, little is known about the mode of action of this phytochemical with bionematicidal properties.

In recent years, advances in omics technologies have opened new possibilities to understand the molecular mechanisms of how control agents act on their targets. For example, *M. incognita* transcriptional changes in response to next‐generation non-fumigant nematicides fluensulfone, fluopyram, fluazaindolizine, and oxamyl were recently assessed and main cellular pathways affected by these compounds were identified (Hada et al., 2021; Wram et al., 2022a; Wram et al., 2022b). Significant downregulation of all neuropeptidergic genes, with concomitant repression of majority of genes related to chemosensation, esophageal gland secretion, parasitism, fatty acid metabolism, and G-protein-coupled receptors, was identified in fluensulfone-treated *M. incognita* J2 (Hada et al., 2021). Also, *M. incognita* transcriptional changes resulting from the exposure to huperzine A, produced by the fungus *Paecilomyces tenuis*, and to a *P. tenuis* filtrate were evaluated, unraveling the molecular mechanisms underlying the nematicidal action of these biocompounds, such as downregulation of genes involved in neural response, signaling, and longevity regulating pathways (Kassam et al., 2023). In another study, transcriptome analysis has shown that the nematicidal compound octanoic acid produced by microorganisms such as *Bacillus altitudinis* and *Megasphaera hexanoica* can interfere with *M. incognita* energy metabolism, lifespan, and signaling, with the cuticle, lysosomes, and extracellular regions and spaces being the primary targets by this compound (Wang et al., 2023).

In the present study, the transcriptome profile of *M. luci* J2, the infective stage, in response to 1,4-NTQ exposure was determined by RNA-seq to identify genes and pathways that might be involved in 1,4-NTQ’s mode(s) of action against this RKN.

## 2 Materials and methods

### 2.1 Nematodes and 1,4-NTQ treatment

1,4-NTQ (purity ≥96.5% w/w, Sigma-Aldrich) was dissolved in an aqueous solution of Tween^®^ 80 (suitable for cell culture, Sigma-Aldrich) at 100 ppm, to get a final 1,4-NTQ concentration of 20 ppm. This concentration has a sub-lethal effect on *M. luci* J2, as previously demonstrated by Maleita et al. (2022). Approximately 18,000 J2 were incubated at 22°C for 3 days in 20 ppm 1,4-NTQ (N treatment). Water (H treatment) and Tween 80^®^ 100 ppm (T treatment) were included as controls. Each of the three treatments was replicated three times. After N and T treatments, J2 were centrifuged to remove 1,4-NTQ and Tween^®^ 80, washed three times with RNase-free water, and stored at −80°C until RNA extraction. Nematodes exposed to H treatment were also subjected to the same procedure.

### 2.2 Total RNA extraction

Nematode RNA was extracted from the nine treated samples by Trizol/chloroform (Invitrogen) extraction and DirectZol clean up (Zymo-research) and treated with the TURBO^™^ DNase (Invitrogen) to remove the remaining DNA, according to the manufacturer’s instructions. Total RNA quality was assessed by RIN (RNA Integrity Number) determination with the Agilent RNA 6000 Pico Assay Kit on the Bioanalyzer 2100 (Agilent Technologies) and quantified by Qubit RNA HS Assay kit on the Qubit 2.0 Fluorometer (Thermo Fisher Scientific). Extracted RNA was stored at −80°C until sequencing or RT-qPCR.

### 2.3 Library preparation and sequencing

A poly(A) enriched strand-specific library was generated with the TruSeq Stranded mRNA sample preparation kit (Illumina) from approximately 0.5 µg of high-quality total RNA of each sample. Transcriptome sequencing was performed on a NextSeq 550 Illumina sequencer with the NextSeq 500/550 High Output v2.5 Reagent Kit (150 cycles), according to the manufacturer’s instructions (Illumina) at Genoinseq (Cantanhede, Portugal).

### 2.4 Data processing

Sequence data were processed at Genoinseq. Raw reads were extracted from the Illumina Nextseq^®^ System in fastq format with bcl2fasta version 2.20.0.422 (Illumina) and quality-filtered with fastp version 0.20.0 (Chen et al., 2018) to trim bases with an average quality lower than Q25 in a window of 5 bases and to remove polyadenylated tails above 10 bases and reads with less than 50 bases. The ribosomal RNA content was estimated with SortMeRNA version 2.1 (Chen et al., 2018). High-quality reads were *de novo* assembled into transcripts using Trinity version 2.2.0 (Grabherr et al., 2011). Reads from each sample were aligned to the *de novo* transcriptome assembly with Trinity’s Bowtie module.

Transcripts were translated into amino acid sequences with Transdecoder version 5.5.0 (TransDecoder, 2021), with parameters set to a minimum length of 50 amino acids and no strand specificity. Translated sequences were then annotated using Diamond version 2.0.8 (Buchfink et al., 2015) against SwissProt and RefSeq with an e-value of 0.001.

### 2.5 Transcript abundance and differentially expressed genes identification and annotation

Transcript abundance and gene count matrix were estimated using Trinity’s Transcript Quantification module with RSEM (Li and Dewey, 2011). Pre-processing and exploratory data analysis (Heatmap, K-means and PCA) of gene-level read count matrix were performed in IDEP.95 (Ge et al., 2018) with default parameters. Differentially expressed genes (DEGs) between conditions were identified using the DESeq2 package in iDEP.95, considering a fold change |log_2_(FC)| ≥ 1 with a false discovery rate (FDR) ≤ 0.05 (adjusted *p*-value).

The gene ontology (GO) annotations of translated transcripts corresponding to DEGs were achieved with OmicsBox (2019) based on the BLAST against the non-redundant protein database NCBI and InterPro database using the default settings in each step (Götz et al., 2008). The GO analysis is done in three categories: (i) molecular function, which defines molecular activities of gene products; (ii) cellular component, which describes where gene products are active; and (iii) biological process, which clarifies the pathways and larger processes made up of the activities of multiple gene products. GO enrichment analysis was conducted for the selected DEGs in a condition against the total of DEGs among the three conditions, in OmicsBox with the statistical Fisher’s Exact Test, using a *p*-value of 0.05 as cutoff. Functional annotation of selected DEGs was complemented with analysis on the Kyoto Encyclopedia of Genes and Genomes (KEGG) pathway database (Kanehisa and Goto, 2000) in OmicsBox.

### 2.6 RT-qPCR analysis of selected DEGs

From the non-overlapping DEGs found in N–H comparison, four genes were selected from the list of enriched GO terms or most representative KEGGs to validate relative quantitative data obtained from sequencing data. The relative transcript abundance of selected genes was assessed by reverse transcription quantitative real-time PCR (RT-qPCR) with SsoAdvance Universal SYBRGreen supermix (Bio-Rad), according to standard protocols, using the CFX96 Touch™ Real-Time PCR Detection System (Bio-Rad). Extracted RNA was converted into cDNA using the iScript cDNA Synthesis kit (Bio-Rad) and used in qPCR. The amplification kinetics of each transcript was normalized with the amplification kinetics of the malate dehydrogenase (*mdh*) and ribosomal protein S6 (*rps6*) genes, chosen as endogenous controls according to Wu et al. (2019) and amplified with primers adapted to *M. luci* corresponding transcript sequences obtained in this study. Other primers used in qPCR were also designed based on *M. luci* transcript sequences (**Table S1**). qPCRs were done at 98°C for 30 s, followed by 40 cycles of 98°C for 5 s and 60°C for 30 s. Melting curve analyses were performed and validation experiments were first carried out to ensure equivalent amplification efficiency for all transcripts. The RT-qPCRs were conducted for the three repetitions of each treatment, with three technical replicates for each qPCR. Amplification efficiencies and Ct values were determined by the CFX ManagerTM Software 2.1 (Bio-Rad) and the mean Ct values used in the REST software (Pfaffl et al., 2002) for relative transcript level and statistically significant differences analysis using the Pairwise Fixed Reallocation Randomization Test^©^.

## 3 Results and discussion

### 3.1 *De novo* transcriptome assembly and transcripts abundance of *M. luci* J2

Sequencing of the nine *M. luci* RNA samples produced 722,024,674 high-quality reads, consisting approximately of a total of 55.5 Gbases. The sample library had an average size of 82,298,380 paired reads and the percentage of high-quality reads after quality filtering was >97% (**Table S2**). From this, *de novo* assembly generated 58,042 transcripts corresponding to the *de novo* transcriptome of *M. luci* J2 available through the Short Read Archive (SRA) under the BioProject accession number PRJNA940699, which constitutes the first transcriptomic data available for this species.

From the 58,042 transcripts, 46,271 were predicted to be translated into amino acid sequences, and from these, 19,393 (33.4%) and 11,663 (20.1%) were annotated against the Swiss-Prot and RefSeq databases, respectively. The low number of annotated transcripts is in accordance with what is found in other PPN species with a significant number of candidate genes lacking annotation and a predicted function (Vieira et al., 2015; Petitot et al., 2016). Compared to genome data on other *Meloidogyne* species, the number of coding transcripts is close to that described for *M. incognita* BioProject PRJEB8714, for which a total of 43,718 coding genes were predicted (Blanc-Mathieu et al., 2017).

**Figure 1:**
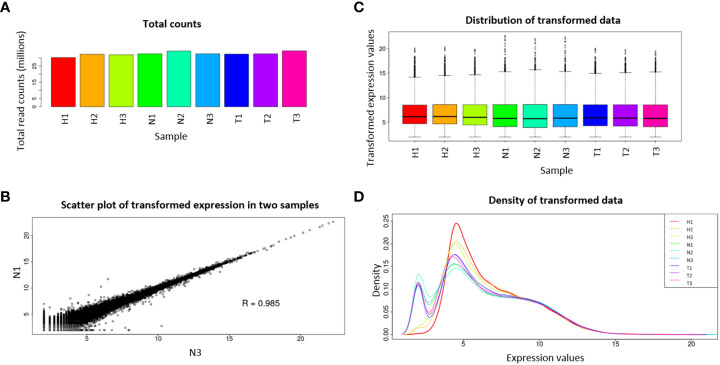
Pre-process diagnostic plots for read count data. Total read counts per library sample **(A)**; scatter plot of N1 and N3 samples **(B)**; boxplot of transformed data **(C)**; distribution of transformed data using a density plot **(D)**. Water (H), 1,4-naphthoquinone (N), and Tween 80^®^ (T) treatments, each with three replicates.

Considering that some of the transcripts correspond to isoforms of the same gene, a gene abundance matrix with a total of 47,435 expected counted genes was obtained, and from these, after IDEP.95 default filtering (at least 0.5 counts per million in at least one library), 28,165 genes were left. Pre-process analysis revealed homogeneity among the nine libraries, with small variation in library sizes (**Figure 1A**), small variation of transformed data between replicates (**Figure 1B**), and similar distribution of the transformed data (**Figures 1C, D**).

**Figure 2:**
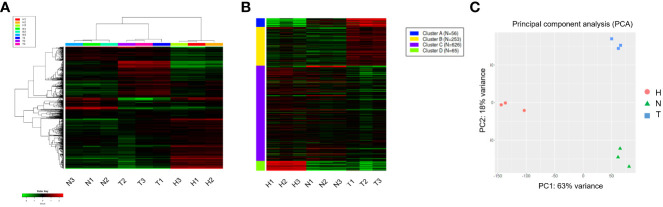
Hierarchical clustering and PCAs of read count data. Heatmap analysis **(A)** and K-means **(B)** analyses of the top 1,000 most variable genes and PCA plot using the first and second principal components **(C)**. Water (H), 1,4-naphthoquinone (N), and Tween 80^®^ (T) treatments, each with three replicates.

The top 1,000 most variable genes were ranked by their standard deviation across all samples in hierarchical clustering and consistent clusters were obtained in Heatmap and K-means analysis (**Figures 2A, B**). A PCA plot using the first and second principal components revealed a clear difference between the water (H) control treatment, 1,4-NTQ (N) treatment, and Tween 80^®^ (T) treatment (**Figure 2C**). Variations among replicates were minimal (**Figure 2**).

### 3.2 Identification of DEGs in response to treatments

**Figure 3:**
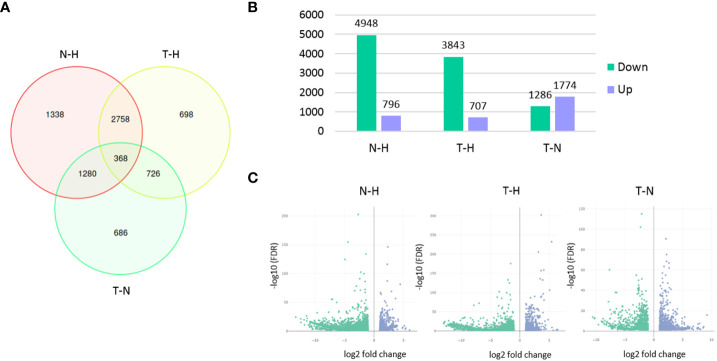
Differentially expressed genes (DEGs) among treatments. Venn diagram presenting the number of unique and overlapping DEGs among the three pairwise comparisons **(A)**, bar chart presenting the number of genes up- or downregulated in each pairwise comparison **(B)**, and volcano plots **(C)** of DEGs analysis in each pairwise comparison. Pairwise comparisons: N–H (1,4-naphthoquinone-water); T–H (Tween 80^®^-water); T–N (Tween 80^®^-1,4-naphthoquinone).

From the 28,165 genes, 7,854 were found differentially expressed among the three conditions. A higher number of DEGs was found between N–H treatments (5,744) than between T–H (4,550) or T–N (3,060) treatments. As expected, a lower number of DEGs was found between T–N treatments, since Tween^®^ 80 (T treatment) was used as the 1,4-NTQ solvent (N treatment). The number of downregulated genes was much higher in both N and T treatments compared to H treatment than the number of upregulated genes in the same comparisons. In the T–N comparison, the differences in the number of up- or downregulated genes were the lowest (**Figure 3**).

Overall, the *M. luci* gene expression was highly influenced by 1,4-NTQ treatment with a higher number of DEGs (5,744) than those reported for *M. incognita* treated with fluensulfone (1,831), fluazaindolizine (3,256), oxamyl (334), and fluopyram (166) (Wram et al., 2022b) or in *M. incognita* treated with *P. tenuis* huperzine A (1,079) or fungal filtrate (1,635) (Kassam et al., 2023).

From the 1,338 non-overlapping DEGs found in the N–H comparison (**Figure 3A**), 250 were found to be upregulated and 1,088 were found to be downregulated (**Table S3**). These genes were further explored as their regulation could be associated with a more specific effect of the 1,4-NTQ.

### 3.3 Functional features of selected DEGs in response to 1,4-NTQ treatment

In order to find which group of genes are overrepresented in the 1,338 DEGs unique in the N–H comparison, a GO enrichment analysis was done for the 250 upregulated and 1,088 downregulated genes against all DEGs. Among the upregulated genes, these analyses revealed a strong enrichment on ABC-type transporter activity, binding, and enzyme regulator activities, belonging to GO molecular function terms, and regulation of biological process and biological regulation, belonging to GO biological process terms (**Table 1**).

**Table 1:** Top 10 enriched gene ontology (GO) terms in the 250 selected upregulated genes.

GO ID	GO name	GO level	GO category	FDR	*p*-value
**GO:0140359**	ABC-type transporter activity	4	MF	1.88E-04	3.12E-08
**GO:0050789**	Regulation of biological process	2	BP	1.92E-04	9.56E-08
**GO:0065007**	Biological regulation	2	BP	1.92E-04	8.83E-08
**GO:0042393**	Histone binding	4	MF	8.36E-04	1.11E-06
**GO:0060589**	Nucleoside-triphosphatase regulator activity	4	MF	8.36E-04	7.72E-07
**GO:0070577**	Lysine-acetylated histone binding	5	MF	8.36E-04	1.11E-06
**GO:0140033**	Acetylation-dependent protein binding	5	MF	8.36E-04	1.11E-06
**GO:0030695**	GTPase regulator activity	5	MF	8.36E-04	7.72E-07
**GO:0047117**	Enoyl-[acyl-carrier-protein] reductase (NADPH, A-specific) activity	6	MF	1.33E-03	5.41E-06
**GO:0030234**	Enzyme regulator activity	3	MF	1.33E-03	3.49E-06

MF, molecular function; BP, biological process; FDR, false discovery rate. Enrichment analysis was performed against the 7,854 differentially expressed genes among the three conditions using the statistical Fisher’s exact test and the p-value of 0.05 as cutoff.

**Figure 4:**
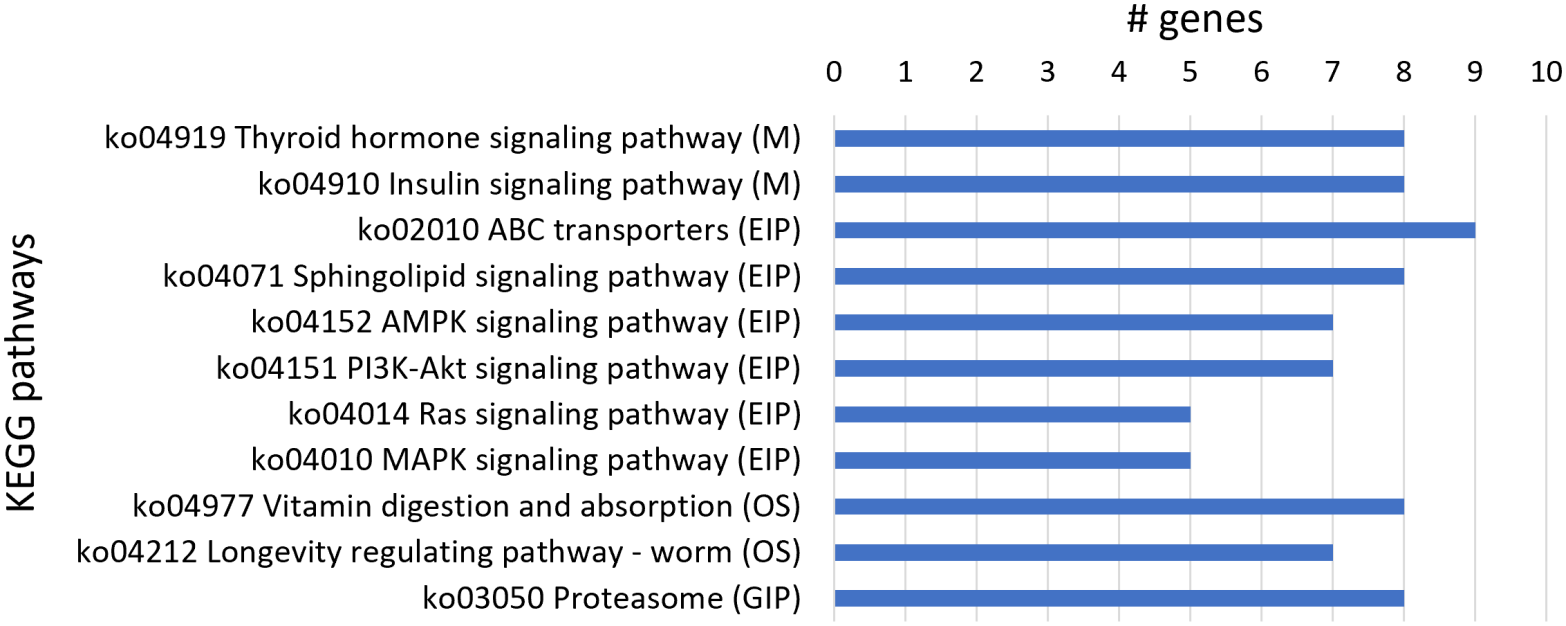
Most represented Kyoto Encyclopedia of Genes and Genomes (KEGG) pathways in the 250 upregulated genes. Number of upregulated transcript sequences associated with KEGG pathways in Metabolism (M), Environmental Information Processing (EIP), Organismal Systems (OS), and Genetic Information Processing (GIP).

From the 250 upregulated genes, 82 transcript sequences were associated with various KEGG pathways (**Table S4**) with a higher number of transcript sequences assigned to the thyroid hormone signaling pathway and insulin signaling pathway in the Metabolism category; ABC transporters, sphingolipid signaling, AMPK signaling, PI3K-Akt signaling, Ras signaling, and MAPK signaling pathways in the Environmental Information Processing category; vitamin digestion and absorption and longevity regulating pathway-worm in the Organismal Systems category; and proteasome in the Genetic Information Processing category. A higher number of transcript sequences of upregulated genes were found to be associated with KEGG pathways belonging to the Environmental Information Processing category, namely, ABC transporters associated with nematode xenobiotic detoxification (**Figure 4**).

On the other hand, among the downregulated genes, an enrichment of GO terms associated with molecular transducer activity and adenylyltransferase and molybdopterin molybdotransferase activities, belonging to the GO molecular function terms, and associated with the molybdopterin cofactor metabolic process and prosthetic group metabolic process, belonging to GO biological process terms, was found (**Table 2**). Molybdopterin cofactor is essential for the catalytic activity of some enzymes such as sulfite oxidase and xanthine dehydrogenase, whose failure is associated with severe neurological abnormalities (Duran et al., 1980), and aldehyde oxidase, which plays an important role in the metabolism of several drugs due to its involvement in cellular redox stress (Garattini and Terao, 2012).

**Table 2:** Top 10 enriched gene ontology (GO) terms in the 1,088 selected downregulated genes.

GO ID	GO name	GO level	GO category	FDR	*p*-value
**GO:0004888**	Transmembrane signaling receptor activity	4	MF	6.84E-08	1.14E-11
**GO:0038023**	Signaling receptor activity	3	MF	2.12E-07	1.06E-10
**GO:0060089**	Molecular transducer activity	2	MF	2.12E-07	1.06E-10
**GO:0004930**	G protein-coupled receptor activity	5	MF	2.79E-07	1.85E-10
**GO:0070566**	Adenylyltransferase activity	6	MF	3.28E-07	2.73E-10
**GO:0043545**	Molybdopterin cofactor metabolic process	4	BP	7.11E-07	1.42E-09
**GO:0051189**	Prosthetic group metabolic process	3	BP	7.11E-07	1.42E-09
**GO:0061598**	Molybdopterin adenylyltransferase activity	7	MF	7.11E-07	1.42E-09
**GO:0061599**	Molybdopterin molybdotransferase activity	4	MF	7.11E-07	1.42E-09
**GO:0006777**	Mo-molybdopterin cofactor biosynthetic process	6	BP	7.11E-07	1.42E-09

MF, molecular function; BP, biological process; FDR, false discovery rate. Enrichment analysis was performed against the 7,854 differentially expressed genes among the three conditions using the statistical Fisher’s exact test and the p-value of 0.05 as cutoff.

**Figure 5:**
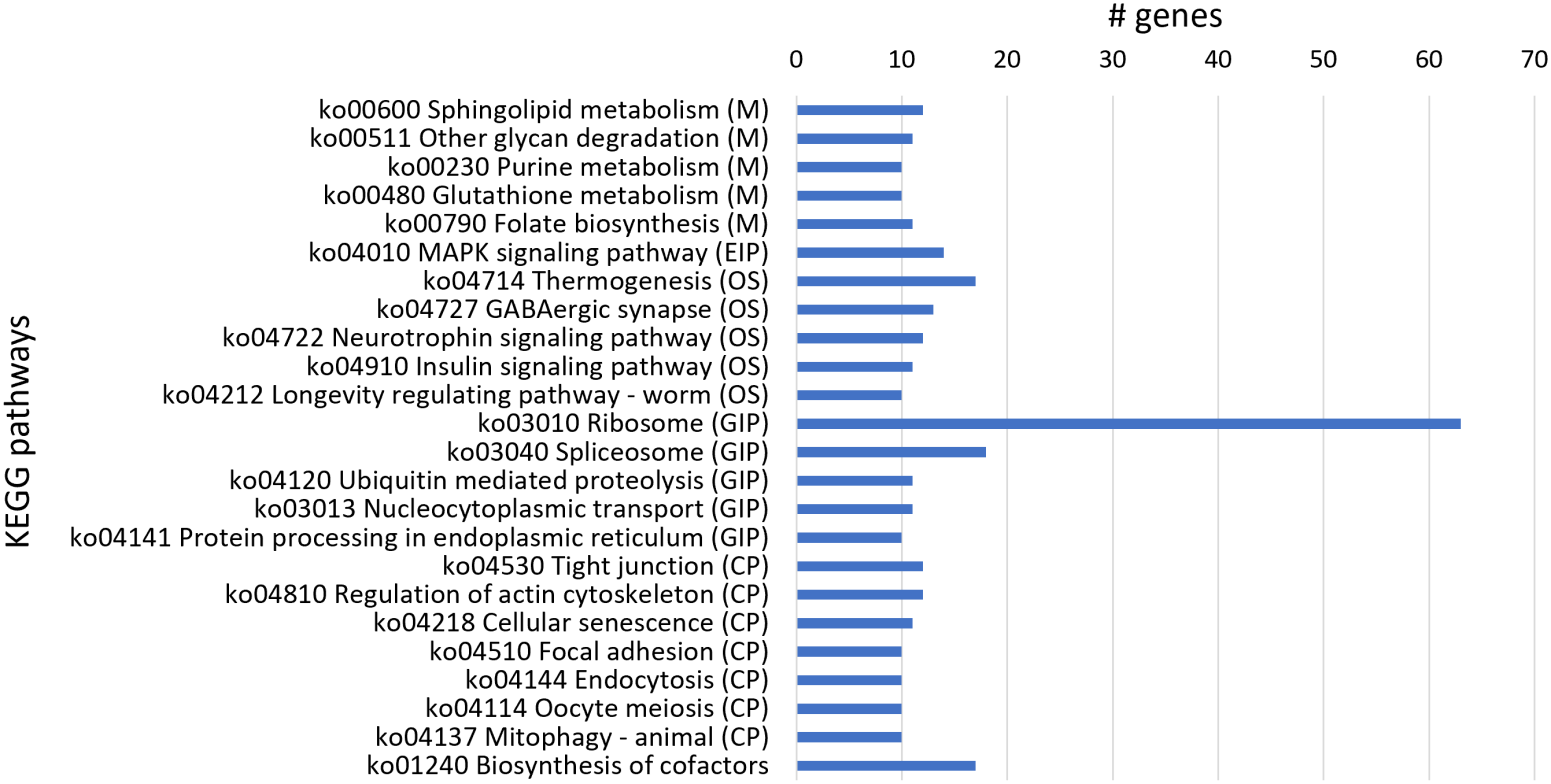
Most represented Kyoto Encyclopedia of Genes and Genomes (KEGG) pathways in the 1,088 downregulated genes. Number of downregulated transcript sequences associated with each KEGG pathway in Metabolism (M), Environmental Information Processing (EIP), Organismal Systems (OS), Genetic Information Processing (GIP), and Cellular Processes (CP).

From KEGG analysis of 1,088 downregulated genes, 316 transcript sequences were associated with various KEGG pathways (**Table S5**), and from these, a higher number of transcript sequences were associated with ribosome and spliceosome in the Genetic Information Processing KEGG category, related to translation and transcription processes, respectively (**Figure 5**). The considerable higher number of downregulated transcripts associated with ribosome reveals a clear inhibitory effect of 1,4-NTQ on translational processes, compromising protein synthesis and consequently several core biological processes. Ribosomes are attractive targets in the antitumor, antiviral, antibacterial, antifungal, and antiparasitic therapies, with several compounds suppressing protein biosynthesis in eukaryotic cells, such as antibiotics, being widely used in medicine and also in agriculture (Lin et al., 2018; Mann et al., 2021). Downregulation of a high number of genes related to ribosome pathway suggests ribosome as one of the main targets of 1,4-NTQ in *M. luci*.

The biosynthesis of cofactors pathway was also highly represented in downregulated genes’ KEGG analysis and is in accordance with molybdopterin cofactor-related activities identified in GO enrichment analysis. Additionally, the thermogenesis pathway, implicated in stress response and ensuring the normal cellular and physiological function under conditions of environmental challenge, was also compromised by 1,4-NTQ, with a high number of downregulated genes associated with this pathway. The neurotoxicity of 1,4-NTQ was also evident with the highly represented GABAergic synapse pathway among the downregulated genes.

Moreover, sphingolipid metabolism is also highly represented among the downregulated genes; however, the related sphingolipid signaling pathway was also identified among the most represented KEGGs in upregulated genes. Sphingolipids are bioactive lipid molecules found in the membranes of all eukaryotic cells and have critical functions in the control of cell growth, senescence, differentiation, and programmed cell death. Different intermediates of sphingolipid pathways can have opposing effects on cell signaling (Haughey, 2010), and therefore, imbalances in sphingolipid metabolism and signaling, caused by 1,4-NTQ, can deregulate key cellular processes in *M. luci*.

Furthermore, in common with most represented KEGGs in upregulated and downregulated genes was the mitogen-activated protein kinase (MAPK) signaling pathway. The MAPK signaling pathway is known to serve as a transducer of extracellular stimuli that allow cellular adaptation to changes in the environment, playing important roles in complex cellular processes like proliferation, differentiation, development, transformation, and apoptosis (Andrusiak and Jin, 2016), and consequently, deregulation of this pathway is another key adverse effect of 1,4-NTQ in *M. luci*.

On the other hand, the most represented KEGG pathway in upregulated genes, ABC transporters, was not identified in downregulated genes. Moreover, the most represented KEGG in downregulated genes, ribosome, was also not identified among the upregulated genes, reflecting a consistent stimulant and inhibitory effect of 1,4-NTQ on ABC transporters and ribosome pathways, respectively.

Overall, it is perceptible that the antagonist effect of 1,4-NTQ on *M. luci* transcriptome disturbs several fundamental cellular processes. These results support the findings in previous bioassays, where 1,4-NTQ showed nematicidal effects on *M. luci* mortality, hatching, root penetration, and reproduction in tomato (Maleita et al., 2022).

### 3.4 DEGs related to nematode xenobiotic detoxification

Nematodes use detoxification pathways to protect themselves from the effect of toxic compounds. Nematode xenobiotic metabolism includes three phases. In phase I, functional groups such as hydroxyl groups are added to xenobiotics by enzymes mainly from cytochrome P450 (CYP) and short-chain dehydrogenase/reductase (SDR) families, to increase their solubility. In a second phase, enzymes mainly from UDP-glucuronosyl transferase (UGT) and glutathione S-transferase (GST) classes catalyze the conjugation of xenobiotics with polar molecules, promoting their water solubility. Finally, the excretion of xenobiotics (phase III) is made by ATP-binding cassette (ABC) transporters and other transmembrane transporters that actively export catalyzed compounds across the cytoplasmic membrane (Hartman et al., 2021).

Transcript levels of enzymes involved in detoxification were clearly affected in *M. luci* J2 exposed to 1,4-NTQ (**Table 3**). From the DEGs belonging to the main enzyme families involved in phase I of detoxification, two CYP were found to be downregulated and one SDR was found to be upregulated. Moreover, from the seven DEGs associated with the metabolism of xenobiotics by cytochrome P450 (ko00980), two were found to be upregulated (one glycosyl hydrolase and one UGT) and five were found to be downregulated (two UGTs, one GST, and two dehydrogenases), compromising phase I and phase II of the detoxification process and possibly compromising the ability to encounter this external toxic compound. In addition, and as discussed above, from GO enrichment analysis, the activity of several enzymes that could be involved in this detoxification process, such as aldehyde oxidase, could be affected not at the transcript level but by their cofactor’s availability. On the other hand, phase III of the detoxification process was stimulated with the upregulation of all transcripts related to ABC transporters and the activation of the final excretion of metabolized toxic compounds. The same tendency was found in the pinewood nematode (PWN), *Bursaphelenchus xylophilus*, in response to emamectin benzoate, used to control PWN infection by pine tree trunk injection, with the downregulation of several UGTs and some of the differentially expressed GSTs and the upregulation of ABC transporters (Lu et al., 2020). Also in *B. xylophilus*, the response to the tree host-derived α- or β-pinene in reaction to pathogen attack resulted in the downregulation of GSTs and the upregulation of ABC transporters, but UGTs were mainly upregulated (Li et al., 2019; Li et al., 2020). In *M. incognita*, transcripts related to xenobiotic detoxification were also revealed to be affected by several nematicides; however, the pattern of up- and downregulated expression varied among the several tested nematicides (Wram et al., 2022b).

**Table 3:** Differentially expressed genes associated with main enzyme families involved in detoxification.

Transcript code	Description	KEGG code/InterPro entry	Log2(FC) N–H
**TRINITY_DN22169_c3_g1**	CYP	-/IPR001128	−1.05
**TRINITY_DN13780_c0_g1**	CYP	-/IPR001128	−1.31
**TRINITY_DN11873_c0_g1**	SDR	-/IPR002347	1.06
**TRINITY_DN17767_c2_g1**	Glycosyl hydrolase	ko00980/IPR000602	1.46
**TRINITY_DN21307_c2_g1**	UGT	ko00980/IPR002213	1.03
**TRINITY_DN19639_c0_g1**	UGT	ko00980/IPR002213	−1.02
**TRINITY_DN21908_c2_g1**	UGT	ko00980/IPR002213	−1.28
**TRINITY_DN29173_c0_g1**	GST	ko00980/IPR004045	−1.91
**TRINITY_DN33953_c0_g1**	Alcohol dehydrogenase	ko00980/IPR013149	−1.83
**TRINITY_DN16092_c0_g1**	aldehyde dehydrogenase	ko00980/IPR015590	−1.88
**TRINITY_DN20723_c2_g1**	ABC transporter	ko02010/IPR036640	1.64
**TRINITY_DN20723_c3_g1**	ABC transporter	ko02010/IPR003439	1.09
**TRINITY_DN20723_c4_g1**	ABC transporter	ko02010/IPR003439	1.11
**TRINITY_DN20723_c5_g1**	ABC transporter	ko02010/IPR027417	1.47
**TRINITY_DN20723_c6_g1**	ABC transporter	ko02010/IPR036640	1.75
**TRINITY_DN19007_c0_g1**	ABC transporter	ko02010/IPR036640	1.13
**TRINITY_DN5013_c1_g1**	ABC transporter	ko02010/-	1.27

CYP, cytochrome P450; SDR, short-chain dehydrogenase/reductase; UGT, UDP-glucuronosyl transferase; GST, glutathione S-transferase; KEGG, Kyoto Encyclopedia of Genes and Genomes; -, not predicted.

### 3.5 Transcription level of selected DEGs

**Figure 6:**
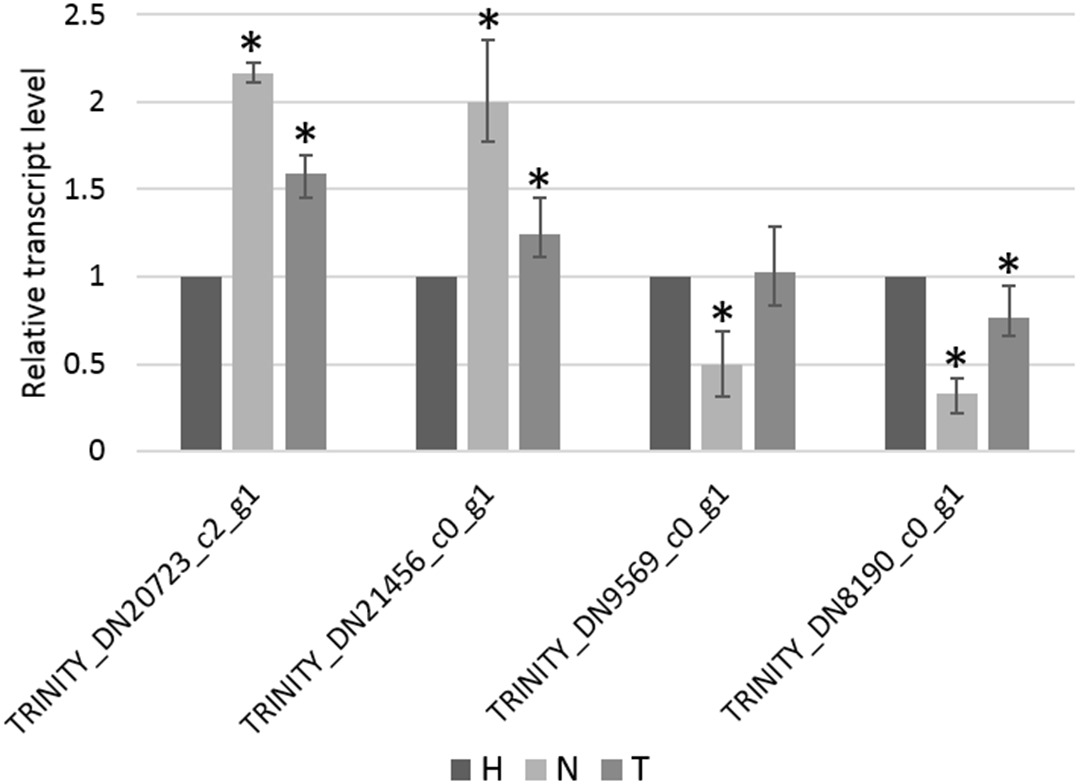
Relative transcript level of TRINITY_DN20723_c2_g1, TRINITY_DN21456_c0_g1, TRINITY_DN9569_c0_g1, and TRINITY_DN8190_c0_g1 measure by RT-qPCR. Bars represent the standard error range of three replicates and asterisks indicate statistically significant differences (*p* < 0.05) between 1,4-naphthoquinone (N) or Tween^®^ 80 (T) treatments and water (H) control, determined using the Pairwise Fixed Reallocation Randomization Test in REST software.

The relative transcript level of four genes, selected from the list of non-overlapping DEGs in the N–H comparison, was determined by RT-qPCR. From these, TRINITY_DN20723_c2_g1 and TRINITY_DN21456_c0_g1 were found to be significantly upregulated in the N-treated condition compared to the H-treated condition, whereas TRINITY_DN9569_c0_g1 and TRINITY_DN8190_c0_g1 were found to be significantly downregulated (**Figure 6**), in accordance with relative quantitative data of those genes obtained from RNA-seq (**Table S6**). Additionally, the pattern of changes in transcript levels of these four genes, among the other two pairwise comparisons (T–H and N-T), was consistent using both qPCR and RNA-seq approaches (**Figure 6**, **Table S6**).

## 4 Conclusions

This study provided the first data on *M. luci* transcriptome and the overall understanding of transcriptional response of this species to 1,4-NTQ exposure, contributing to an increased knowledge of how this bionematicidal compound interacts with nematodes. The newly assembled transcriptome represents an important resource for our understanding of the biology of this RKN and can be used for applications, such as gene discovery or comparison of *M. luci* transcriptome with other PPNs, to understand the molecular origin of their parasitism. High transcriptional effects were detected on *M. luci* with a high number of DEGs found between 1,4-NTQ treatment and water control. From these, a much higher number of downregulated genes than upregulated genes were identified, reflecting the inhibitory effect of 1,4-NTQ on *M. luci*. This inhibitory effect had a great impact on processes related to translation (ribosome pathway) and probably be even more evident at the protein level than at the transcript level. Further research on the proteomics of *M. luci* and other RKN species exposed to 1,4-NTQ would be important to clarify the post-transcriptional effects of this compound.

## Data availability statement

The datasets presented in this study can be found in online repositories. The names of the repository/repositories and accession number(s) can be found below: https://www.ncbi.nlm.nih.gov/sra/PRJNA940699.

## Author contributions

JC, IE, CE, IA and CM conceived and designed the experiments. JC, IE, MB and CM performed the experiments. JC, CE, IE and CM analyzed the data. JC wrote the original draft. HdS and CM acquired the funding. All authors contributed to the article and approved the submitted version.
